# Pathogenesis of and strategies for preventing *Edwardsiella tarda* infection in fish

**DOI:** 10.1186/1297-9716-43-67

**Published:** 2012-10-04

**Authors:** Seong Bin Park, Takashi Aoki, Tae Sung Jung

**Affiliations:** 1 Aquatic Biotechnology Center, College of Veterinary Medicine, Gyeongsang National University, Jinju, 660-701, South Korea; 2 Consolidated Research Institute for Advanced Science and Medical Care (ASMeW), Waseda University, 513, Wasedatsurumaki-cho, Shinjuku-ku, Tokyo, 162-0041, Japan

## Abstract

*Edwardsiella tarda* is one of the serious fish pathogens, infecting both cultured and wild fish species. Research on edwardsiellosis has revealed that *E. tarda* has a broad host range and geographic distribution, and contains important virulence factors that enhance bacterial survival and pathogenesis in hosts. Although recent progress in edwardsiellosis research has enabled the development of numerous, highly effective vaccine candidates, these efforts have not been translated into a commercialized vaccine. The present review aims to provide an overview of the identification, pathology, diagnosis and virulence factors of *E. tarda* in fish, and describe recent strategies for developing vaccines against edwardsiellosis. The hope is that this presentation will be useful not only from the standpoint of understanding the pathogenesis of *E. tarda*, but also from the perspective of facilitating the development of effective vaccines.

## Table of contents

1. Introduction

2. Identification and classification

3. Hosts

4. Pathology and diagnosis

5. Virulence factors

6. Vaccines

7. Concluding remarks

8. Competing interests

9. Authors’ contributions

10. Acknowledgements

11. References

## 1. Introduction

Edwardsiellosis, caused by *Edwardsiella tarda,* has been reported worldwide in economically important fish species, including Japanese eel (*Anguilla japonica*), red sea bream (*Pagrus major*), yellowtail (*Seriola quinqueradiata*), channel catfish (*Ictalurus punctatus*), and turbot (*Scophthalmus maximus*) [1-4]. This infection also leads to serious economic losses in the aquaculture of olive flounder (Japanese flounder; *Paralichthys olivaceus*), the most important fish species in South Korean aquaculture, with production valued at 489.7 billion Korean Won (40 922 MT), which corresponds to 56.5% of total fisheries production in 2010 [5-7].

Recent studies on vaccine development have applied a variety of antigen-preparation methods; however, commercial vaccines are not yet available. In addition, numerous studies have reported on virulence factors of *E. tarda* and immune responses of hosts. In the present study, the pathogenicities of *E. tarda* in fish that can be exploited to elicit effective protection strategies against edwardsiellosis will be discussed.

## 2. Identification and classification

The genus *Edwardsiella* is composed of three species, *E. tarda*, *E. ictaluri*, and *E. hoshinae *[8-10]. Fish are usually infected with *E. tarda* or *E. ictaluri*, whereas *E. hoshinae* infection is usually reported in reptiles and birds [11]. Panangala et al. [12] suggested that biochemical tests can differentiate *E. tarda* from *E. ictaluri* among bacteria isolated from freshwater fish based on the positive reaction of *E. tarda* in tests of indole production, methyl red reduction and hydrogen sulfide generation. In protein profiling of bacterial isolates using sodium dodecyl sulfate-polyacrylamide gel electrophoresis (SDS-PAGE) and Western blotting, the authors also demonstrated that *E. ictaluri* is more homogenously distributed than *E. tarda*.

*E. tarda* was originally isolated from cultured Japanese eel (*Anguilla japonica*) in Japan in 1962 [1]. Subsequent findings for the bacterium were reported from snakes in Japan [13] and from human feces in the USA; the bacterium was designated *E. tarda* by Ewing et al. [8]. Although there was a move to change the epithet *tarda* to *anguillimortiferum* since it had been initially reported as *Paracolobactrum anguillimortiferum *[14], the bacterium is commonly named *E. tarda* because *P. anguillimortiferum* was not registered and the original culture was lost [6,15].

*E. tarda* is a Gram-negative, short, rod–shaped, facultative anaerobic bacterium that measures about 2–3 μm in length and 1 μm in diameter [11]. It is usually motile, but isolates from red sea bream and yellowtail are non-motile [16]. This bacterium can survive at 0–4% sodium chloride, pH 4.0–10.0, and 14–45°C [17]. The biochemical characteristics of *E. tarda* are catalase positive, cytochrome oxidase negative, production of indole and hydrogen sulfide, fermentation of glucose, and reduction of nitrate to nitrite [11]. However, several variations of biochemical tests have been found for ornithine decarboxylase, citrate utilization, hydrogen sulfide production, and fermentation of mannitol and arabinose. These discrepancies allow division into two groups: wild type and biogroup 1 [8,9,18]. The characteristics of wild-type *E. tarda* are negative for arabinose, mannitol and sucrose production, and positive for hydrogen sulfide production; the characteristics of biogroup 1 are the opposite.

Park et al. [19] demonstrated that *E. tarda* can be divided into four serotypes, A, B, C and D, using O-antigen extracts of 445 isolates from infected eel, water, and sediments. They suggested that 72% of isolates belonged to serotype A, the most virulent group based on experimental challenge tests, whereas a subsequent study of *E. tarda* isolated from olive flounder revealed that all isolates were serotype A [20]. Another study established an *E. tarda* serotyping scheme comprising 61 O groups and 45 H antigens that is preferable for international applications [21].

Several important findings suggest that intra- and/or inter-specific variability exists among *E. tarda* strains. *E. tarda* isolated from humans could be differentiated from isolates from fish by RAPD (random amplified polymorphic DNA) analysis [22], and *E. tarda* isolated from freshwater fish or pond sediments showed diverse and/or homogeneous characteristics in plasmid profiling, ERIC-PCR (enterobacterial repetitive intergenic consensus-polymerase chain reaction), SDS-PAGE, and RFLP (restriction fragment length polymorphism) analyses of 16S rDNA [12,23,24]. In addition, Western blot profiles of LPS (lipopolysaccharides) from *E. tarda* strains isolated from turbot and other fish revealed that only isolates from turbot were recognized by rabbit sera raised against the isolate from turbot [25]. Biochemical tests, protein profiling, LPS profiling, and RAPD analysis showed that *E. tarda* strains from olive flounder have highly homogeneous phenotypic and genotypic characteristics compared to isolates from Japanese eel (unpublished data).

## 3. Hosts

Since the first report of *E. tarda* infection in Japanese eel [1], *E. tarda* has been isolated from numerous marine and freshwater fishes, including barramundi (*Lates calcarifer*) [26], channel catfish [3], largemouth bass (*Micropterus salmoides*) [27], mullet (*Mugil cephalus*) [28], crimson sea bream (*Evynnis japonica*) [29], tilapia (*Tilapia nilotica*) [30], chinook salmon (*Oncorhynchus tshawytscha*) [31], red sea bream [2], yellow tail [2], olive flounder [32], common carp (*Cyprinus carpio*) [33], European sea bass (*Dicentrarchus labrax*) [34], turbot [4], Asian catfish (*Claris batrachus*) [35], brook trout (*Salvelinus fontinalis*) [36], Indian major carp (*Catla catla*) [37], rohu (*Labeo rohita*) [37], European eel (*Anguilla anguilla*) [38], and Far Eastern catfish (*Silurus asotus*) [39]. In addition, isolation of *E. tarda* has been reported in invertebrates [40], amphibians [40], reptiles [13,26], birds [27,41,42] and mammals, including humans, cattle, swine, dogs, and Weddell seals (*Leptonychotes weddellii*) [8,41-45]. These numerous reports indicate that *E. tarda* has a wide geographical distribution, even in Antarctica [42], and is an important pathogen in terms of public health, since it can progress as an epizootic and zoonotic bacterium [11].

## 4. Pathology and diagnosis

**Figure 1:**
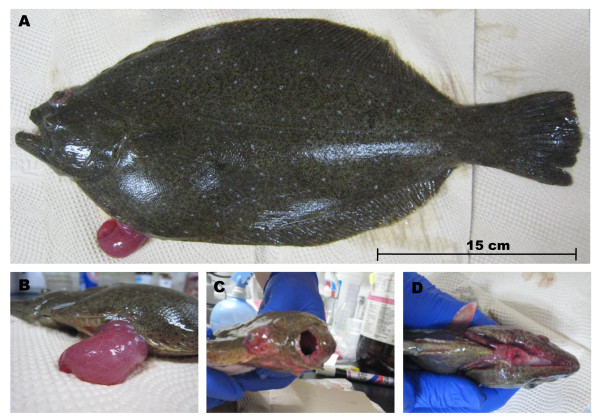
**External signs of olive flounder infected with *Edwardsiella tarda*. A**: External lesions of diseased fish. **B**: Abdominal distension and rectal hernia. **C**: Exophthalmia and opacity of the eye. **D**: Peripheral hyperemia in mandible lesion.

Edwardsiellosis in fish usually occurs under imbalanced environmental conditions, such as high water temperature, poor water quality, and high organic content [11]. Fish infected with *E. tarda* show abnormal swimming behavior, including spiral movement and floating near the water surface [3,46]. Although clinical signs vary after onset, fish infected with *E. tarda* show loss of pigmentation, exophthalmia, opacity of the eyes, swelling of the abdominal surface, petechial hemorrhage in fin and skin, and rectal hernia (Figure 1) [6,35,46]. Internally, watery and bloody ascites in the abdominal space and congested liver, spleen, and kidney are found [46,47]. Histopathological characteristics of edwardsiellosis in fish are suppurative interstitial nephritis, suppurative hepatitis, and purulent inflammation in the spleen [6,46-48]. Abscesses of various sizes, bacterial colonization, and infiltration of neutrophils and macrophages are found in the liver, spleen, and kidney [6,47,48]. Some remarkable pathological features have also been demonstrated in fish, such as dorsolateral petechial hemorrhage and abscesses in cutaneous lesions of channel catfish [3,49]; hyperplasia, necrosis and inflammation in lateral line canals of striped bass [50]; and necrosis and aggregation of bacteria-laden macrophages in red sea bream [46]. However, the symptoms and pathological changes in fish are similar to those of other bacterial infections, including *Aeromonas hydrophila*, *Vibrio anguillarum* and *Pseudomonas anguilliseptica*; thus, other molecular or biochemical methods are recommended for diagnosis of *E. tarda* infection [51].

*E. tarda* is usually identified based on its unique biochemical characteristics after isolation on brain-heart infusion agar or tryptone soya agar from infected fish. Several studies have suggested that serological techniques are useful for diagnosis of *E. tarda* infection, including agglutination tests, enzyme linked immunosorbent assays (ELISA), and fluorescent antibody techniques [3,31,37]. Recently, PCR-based methods have been reported for accurate, sensitive, and differential diagnosis. Real-time PCR has been used to analyze the blood of oyster toadfish (*Opsanus tau*) infected with *E. tarda *[52], and the loop-mediated isothermal amplification (LAMP) method is able to detect *E. tarda* in infected tissue samples and pond water [53]. Chang et al. [54] developed a multiplex nested PCR for four important fish pathogens in subtropical Asia that can simultaneously detect *A. hydrophila*, *E. tarda*, *Photobacterium damselae* and *Streptococcus iniae* from pure colonies and tissue homogenates. In addition, a primer set, evaluated using 53 *E. tarda* strains isolated from various sources and 18 representative strains of related and unrelated bacterial species, was shown capable of detecting two cells from pure culture and 3 × 10^2^ cells in seeded turbot tissues [55].

## 5. Virulence factors

**Figure 2:**
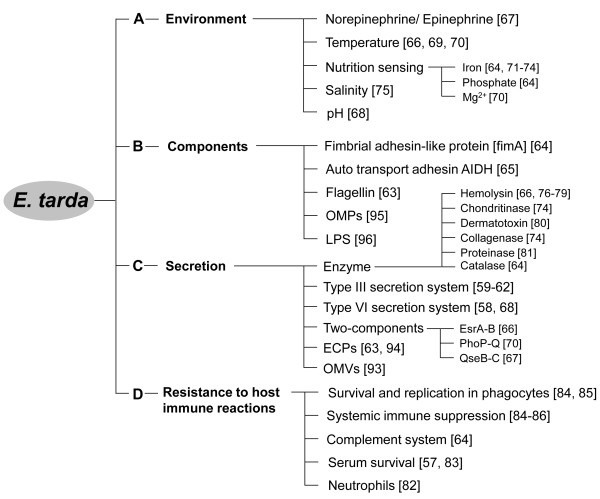
**Factors contributing to *Edwardsiella tarda *survival and infection*. *A**: *E. tarda* can survive by utilizing host and environmental elements. **B** and **C**: *E. tarda* possesses or secretes several virulence factors which may support multifactorial pathogenesis in the host. **D**: *E. tarda* is able to resist against host humoral and cellular immunities. Numbers in the brackets indicate the references.

*E. tarda* survive in their host by utilizing several important substances and abilities that serve as virulence factors in the host (Figure 2). A study using green fluorescent protein (GFP) showed that both avirulent and virulent *E. tarda* are able to adhere to, invade, and replicate in the carp epithelial papilloma (EPC) cell line using host microfilaments and protein tyrosine kinase [56]. Histopathological and infection kinetics studies using GFP revealed that the gill, gastrointestinal tract, and body surface of blue gourami (*Trichogaster trichopterus*) are the sites of entry of the virulent strain [57].

Type ІІІ secretion system (T3SS) and type VІ secretion system (T6SS) play important roles in adherence, penetration, survival, and replication of *E. tarda* in epithelial cells and phagocytes (Table 1). The T6SS of *E. tarda* comprises 16 genes, and 13 of the encoded proteins are involved in the secretion of EvpP (*E. tarda* virulence protein) [58]. Three proteins (EvpP, EvpI and EvpC) are secreted into the extracellular milieu, and the secretion of EvpC and EvpI are required for the secretion of EvpP [58]. The putative ATPase, EvpO, contains a Walker A motif, which possibly interacts with EvpA, EvpL, and EvpN [58]. T3SS is a multi-protein complex that is essential for host and pathogen interaction. The central component of T3SS is a needle complex, which is structurally similar to bacterial flagella, that spans the bacterial inner and outer membrane [59]. This needle can connect to the host cell membrane via the tip complex through the translocon, which can allow the delivery of bacterial effector proteins from an ATPase dependent manner [60]. In *E. tarda*, T3SS proteins include the *E. tarda* secretion system apparatus (EsaB and EsaN), effectors (EseB, EseC and EseD), chaperones (EscA, EscB and EscC), and regulators (EsrA, EsrB and EsrC) [60-62]. Proteomic studies have revealed that EseB, EseC and EseD are the major ECP, and mutations of these genes in *E. tarda* reduces virulence compared to parental *E. tarda*[63].

**Table 1:** The virulence factors described in the present study

**Abbreviation**	**Name**	**Accession number**	**Function**
**Type III secretion systems**			
EsaB	putative TTSS apparatus protein B	AAV69410	apparatus
EsaC	putative TTSS apparatus protein C	AAV69411	apparatus
EsaD	putative TTSS apparatus protein D	AAV69412	apparatus
EsaG	putative TTSS apparatus protein G	AAV69415	apparatus
EsaH	putative TTSS apparatus protein H	AAV69416	apparatus
EsaI	putative TTSS apparatus protein I	AAV69417	apparatus
EsaJ	putative TTSS apparatus protein J	AAX76915	apparatus
EsaK	putative TTSS apparatus protein K	AAX76913	apparatus
EsaL	putative TTSS apparatus protein L	AAV69401	apparatus
EsaM	putative TTSS apparatus protein M	AAX76922	apparatus
EsaN	putative TTSS apparatus protein N	AAX76920	apparatus
EsaQ	putative TTSS apparatus protein Q	AAV69420	apparatus
EsaR	putative TTSS apparatus protein R	AAX76923	apparatus
EsaS	putative TTSS apparatus protein S	AAV69419	apparatus
EsaT	putative TTSS apparatus protein T	AAX76924	apparatus
EsaU	putative TTSS apparatus protein U	AAV69421	apparatus
EsaV	putative TTSS apparatus protein V	AAX76921	apparatus
EscA	putative TTSS chaperone protein A	AAV69403	chaperone
EscB	putative TTSS chaperone protein B	AAX76917	chaperone
EscC	putative TTSS chaperone protein C	AAV69402	chaperone
EseB	putative TTSS effector protein B	AAX76903	effector
EseC	putative TTSS effector protein C	AAV69404	effector
EseD	putative TTSS effector protein D	AAV69405	effector
EseE	putative TTSS effector protein E	AAV69406	effector
EseG	putative TTSS effector protein G	AAX76916	effector
EsrA	TTSS regulator protein A	AAV69423	regulator
EsrB	TTSS regulator protein B	AAX76904	regulator
EsrC	TTSS regulator protein C	AAV69414	regulator
**Type VI secretion systems**			
EvpA	*E. tarda* virulent protein A	AAR83927	apparatus
EvpB	*E. tarda* virulent protein B	AAR83928	apparatus
EvpC	*E. tarda* virulent protein C	AAR83929	extracellular apparatus
EvpD	*E. tarda* virulent protein D	AAR83930	apparatus
EvpE	*E. tarda* virulent protein E	AAS58123	apparatus
EvpF	*E. tarda* virulent protein F	AAS58124	apparatus
EvpG	*E. tarda* virulent protein G	AAS58125	apparatus
EvpH	*E. tarda* virulent protein H	AAS58126	apparatus
EvpI	*E. tarda* virulent protein I	ABW69081	extracellular apparatus
EvpJ	*E. tarda* virulent protein J	ABW69082	apparatus
EvpK	*E. tarda* virulent protein K	ABW69083	apparatus
EvpL	*E. tarda* virulent protein L	ABW69084	apparatus
EvpM	*E. tarda* virulent protein M	ABW69085	apparatus
EvpN	*E. tarda* virulent protein N	ABW69086	apparatus
EvpO	*E. tarda* virulent protein O	ABW69087	apparatus
EvpP	*E. tarda* virulent protein P	ABW69080	extracellular apparatus
**The other proteins**			
AidA	putative autotransporter protein AidA	BAH03175	autotransporter adhesin
HhaEt	α-hemolysin-modulator like protein	YP_003295064	nucleoid-associated proteins
EthA	*E. tarda* hemolysin A	BAA21097	hemolysin
EthB	*E. tarda* hemolysin B	BAA21096	hemolysin activation/secretion
QseB	DNA-binding transcriptional regulator QseB	ADO13165	Quorum sensing (QS) system
QseC	sensor protein QseC	ADO24152	Quorum sensing (QS) system
PhoP	two-component regulator protein PhoP	ADB28435	DNA-binding transcriptional regulator
PhoQ	two-component sensor protein PhoQ	ADB28436	sensor

Several reports found that motility-related proteins, such as flagellin and autotransport adhesin AIDA, a fimbrial adhesin-like protein, are important for attachment and penetration into the epithelial cells of hosts [63-65]. An *E. tarda* mutant containing a deletion of the *eth*A gene (hemolysin gene locus from *E. tarda*), regulated by the two-component system EsrA-EsrB and nucleid protein Hha_Et_, shows reduced capacity to internalize into EPC cells [66]. Interestingly, a recent study showed that the qseB and qseC two-component system of *E. tarda* inhibits flagella biosynthesis and motility, and induces the expression of the T3SS after invasion into host cells [67]. These findings indicate that *E. tarda* is capable of modulating the expression of genes involved in adjusting to environmental changes, such as adaptation to intracellular living.

Indeed, *E. tarda* is able to survive and adapt to various host environmental conditions, including host hormonal change, temperature, pH, salinity, and variations in several important nutritional elements, such as iron, phosphate, and Mg^2+ ^[64-74]. The qseB and qseC two-component system, an important virulence regulator that contributes to intracellular replication and systemic infection, are able to regulate flagella motility and the intracellular expression of T3SS elements EseB and EsaC in response to eukaryotic hormone-like signals, such as epinephrine and norepinephrine [67]. The PhoP-PhoQ two-component system of *E. tarda* is able to sense changes in temperature and Mg^2+^ concentration and control the T3SS and T6SS via activation of *esrB *[70]. This study showed that a conformational change in PhoQ over a temperature range of 23–37°C and at low Mg^2+^ concentration causes PhoQ autophosphorylation and subsequent activation of PhoP, which promotes expression of *esrB* and leads to secretion of virulent proteins, whereas below 20°C or above 37°C, no such conformational change in PhoQ takes place and the production of virulence proteins is decreased. Similarly, on the basis of observations of mutants containing an insertion of the *pstSCAB-phoU* operon, which is part of the phosphate regulon, Srinivasa Rao et al. [64] suggested that natural conditions of low inorganic phosphate in phagocytic and epithelial cells might stimulate virulent genes to promote survival and replication within the host. In another study, a high concentration of NaCl (3%) was shown to induce hemagglutination activity, which correlated with the expression of fimbrial major subunit (FimA), a 19.3 kDa protein; moreover, *E. tarda* enriched for this fimbrial protein showed higher virulence in challenge experiments compared to *E. tarda* raised in 0% NaCl broth [75].

The ability of bacteria to acquire iron acquisition using the bacterial iron chelator, siderophore, is essential for the survival and replication of bacteria [64,73]. A natural mutant with lower siderophore production and a mutant with a gene encoding aryl sulfate sulfotransferase producing less siderophore showed significantly reduced virulence in *E*. *tarda* challenge experiments [72,73]. Recently, an *E. tarda* deletion mutant lacking the T6SS component *evpP*, which encodes a consensus ferric uptake regulator (Fur) box, was shown to exhibit low virulence in vivo and in vitro [74]. This finding might indicate that the EvpP protein in T6SS plays an important role in invasion mechanisms and thus may be a critical virulence factor.

It has been reported that *E. tarda* produces two kinds of hemolysin; one is a cell associated, iron-regulated hemolysin, encoded by *eth*A and *eth*B, that is secreted as an extracellular protein (ECP) under iron-regulated conditions [76], and the other is an extracellular hole-forming hemolysin distinct from EthA and EthB that is not regulated by iron [77-79]. A recent functional study demonstrated that EthA is critical for invasion in vivo and in vitro, and is regulated by the two-component system EsrA-EsrB and nucleid protein Hha_Et _[66]. Other enzymes, including catalase, chondroitinase, dermatotoxin, protease, and collagenase, are also important for the pathogenesis of *E. tarda*[64,74,80,81].

Several studies have indicated that *E. tarda* is able to survive and replicate in phagocytes, leading to systemic infections [82-86]. Virulent *E. tarda* opsonized with serum of blue gourami can replicate within phagocytes and fails to induce an oxidative burst, possibly providing a mechanism for avoiding phagocyte-mediated killing [83]. A subsequent study revealed that the expression of the catalase (*Kat*B) gene of *E. tarda* is responsible for the resistance to H_2_O_2_ and phagocyte-mediated killing [64]. Similarly, a comparison of the response of peritoneal macrophages from olive flounder to high- and low-virulence *E. tarda* demonstrated that only the highly virulent strain is able to resist reactive oxygen species generated by macrophages, and survive and replicate within macrophages [84]. In a subsequent report, the authors of this latter study extended their results, demonstrating that virulent *E. tarda* elicit a significantly greater induction of nitric oxide and tumor necrosis factor (TNF)-α production by macrophages, actions that may account for the pathogenicity of *E. tarda* infection [85]. In addition, a study of *E. tarda* septicemia revealed that *E. tarda* induces systemic immunosuppression through lymphocyte apoptosis, which suppresses systemic immune responses during the initial stage of septicemia [86].

The host also seems to possess immune mechanisms for avoiding or resisting the propagation of *E. tarda*. An examination of the pathogenicity of motile and non-motile *E. tarda* strains toward olive flounder, red sea bream, and yellow tail showed that all strains were virulent in the olive flounder and yellow tail, whereas only atypical strains showed mortality in the red sea bream [16]. These findings might indicate that immune mechanisms involved in recognition of and resistances against *E. tarda* vary among hosts. In zebra fish (*Danio rerio*), experimental infection with *E. tarda* resulted in an acute elevation of the inflammatory cytokines, interleukin-1β (IL-1β) and TNF-α [87]. Indian major carp challenged with *E. tarda* exhibited a significant induction of immune responses and expression of several immune related genes, including IL-1β, TNF-α, inducible nitric oxide synthase (iNOS), complement component C3, β_2_-microglobulin, CXCa, and C-type and G-type lysozyme [88]. T. Aoki and colleagues surveyed over a thousand genes in olive flounder infected with *E. tarda* using microarray analyses, identifying 36 genes that were differentially expressed between susceptible and resistant olive flounder groups [89-91]. Notably, 3 days post challenge, MHC class I antigen processing- and presenting-related genes were highly expressed in resistant groups, but susceptible groups showed high expression of genes involved in innate immune responses [90].

Understanding the virulence factors of *E. tarda* may inform the development of protection strategies against edwardsiellosis in fish. Recent progress in analytical methods, such as genomics and proteomics, has revealed important virulence factors, including T3SS, T6SS, and two-component systems (Figure 2). Verjan et al. demonstrated seven antigenic proteins, which were identified as lipoproteins, periplasmic proteins, exported, and secreted proteins [92]. Additional proteomic studies on outer membrane proteins (OMP), ECP, and outer membrane vesicles (OMV) may also contribute to the development of effective protection strategies against edwardsiellosis [60,63,65,69,93-98]. In addition, knowing the full genome sequence of *E. tarda* would enhance our understanding of the relationship between *E. tarda* and the host, and further the development of new prophylactic and therapeutic strategies for managing edwardsiellosis in fish [99].

## 6. Vaccines

A vaccine is by definition a biological preparation that improves immunity to a specific disease. The vaccine typically consists of several agents, such as weakened or killed forms of the microbe, its toxin or one of its surface proteins [100]. Numerous antigen preparation methods have been used to develop effective vaccines against edwardsiellosis, including formalin killed cells (FKC), LPS, ECP, live attenuated *E. tarda*, avirulent *E. tarda*, ghost cells, OMP, recombinant proteins, recombinant protein-expressing cells, OMV, and DNA vaccines (Table 2). Several early studies noted that immunization of Japanese eel with FKC or LPS exerted protective effects after challenge with a virulent strain of *E. tarda *[101-103]. However, another study reported no protective effect of FKC and LPS against *E. tarda* infection [104], possibly indicating the diverse antigenicity of *E. tarda* species [105]. Recent progress in vaccine preparation using diverse antigens has led to highly effective vaccines against *E. tarda* infection [106-122]. Several vaccine trials coupled with adjuvants have shown 100% relative survival, and most evaluated trials were shown to produce significant protective effects (Table 2). Interestingly, more than 75% of the studies published in China, Japan and South Korea during the last decade have focused on the protective effects of vaccines in olive flounder, providing an indication that edwardsiellosis in olive flounder is a serious problem in Far East Asia and highlighting the urgent need to develop an effective, commercializable vaccine. Thus, to improve the efficacy of the vaccine, comprehensive understanding of bacterial pathogenesis, including intracellular surviving, host cell mediated-immune responses, and comparative epidemiological investigation on *E. tarda* originating from different finfish species is necessary. These efforts will allow for the identification of host pathogen cross-talk, which will lead to the identification of valuable vaccine candidates, such as a cocktail bacterin vaccine originating from different fish species, mutant vaccine made by deleting different genes compared with previous studies and DNA vaccine combined with several important multiple antigen genes.

**Table 2:** Vaccine trials using a variety of antigen-preparation methods during the last decade

**No.**	**Antigen**	**Adjuvant**	**fish**	**Route**	RPS (%)*	**Country**	**year**	**Ref.**
1	recombinant vaccine DnaJ	aluminum hydroxide	olive flounder	i.p.	62	China	2011	[106]
2	recombinant vaccine OMP	-	common carp	i.p.	54.3	India	2011	[107]
3	natural OMVs	-	olive flounder	i.p.	70	Korea	2011	[93]
4	*Δalr Δasd E. tarda*	*-*	olive flounder	i.p.	100	Korea	2011	[108]
5	recombinant vaccine rEta2	aluminum hydroxide	olive flounder	i.p.	83	China	2011	[109]
6	DNA vaccine pCEta2	-	olive flounder	i.m.	67	China	2011	[109]
7	recombinant vaccine pCEsa1	-	olive flounder	i.p.	57	China	2011	[110]
8	Esa1-expressing recombinant strain	aluminum hydroxide	olive flounder	PO	52	China	2010	[111]
9	Esa1-expressing recombinant strain	aluminum hydroxide	olive flounder	i.p.	79	China	2010	[111]
10	Live E22	-	olive flounder	i.p.	45	Japan	2010	[112]
11	DNA vaccine N163	-	olive flounder	i.m.	70.2	China	2010	[113]
12	recombinant vaccine scFv	Freund's incomplete adjuvant	Red drum	i.p.	88	China	2010	[114]
13	recombinant vaccine EseD	Freund’s complete adjuvant	turbot	i.p.	62.5	China	2010	[62]
14	recombinant vaccine DegP_Et_	Freund's incomplete adjuvant	olive flounder	i.p.	89	China	2010	[81]
15	Recombinant vaccine Et49	Freund's incomplete adjuvant	olive flounder	i.p.	47	China	2010	[81]
16	Live ATCC 15947	-	olive flounder	i.p.	100	China	2010	[115]
17	Extracted OMP	Freund's incomplete adjuvant	olive flounder	i.p.	71	China	2010	[97]
18	recombinant vaccine Eta21	*Bacillus* sp. strain B187	olive flounder	i.p.	69	China	2009	[116]
19	DH5α/pTAET21	*Bacillus* sp. strain B187	olive flounder	i.p.	100	China	2009	[116]
20	DNA vaccine pEta6	-	olive flounder	i.m.	50	China	2009	[117]
21	recombinant vaccine Eta6	*Bacillus* sp. strain B187	olive flounder	i.p.	53	China	2009	[117]
22	recombinant vaccine Et18	*Bacillus* sp. strain B187	olive flounder	i.p.	61	China	2009	[118]
23	recombinant vaccine EseD	*Bacillus* sp. strain B187	olive flounder	i.p.	51.3	China	2009	[118]
24	Formalin killed ACC35.1	Montanide ISA 763 AVG	turbot	i.p.	100	Spain	2008	[55]
25	live, attenuated *esrB* mutant	*-*	turbot	i.p.	93.3	China	2007	[119]
26	Ghost vaccine	*-*	olive flounder	PO	85.7	Korea	2007	[120]
27	Ghost vaccine	*-*	tilapia	i.p.	88.8	Korea	2006	[121]
28	37 kDa OMP	*-*	olive flounder	i.p.	70	Japan	2004	[122]
29	Formalin killed virulent bacterin	*-*	Indian major carp	immersion	98	India	2002	[37]

“-” means no adjuvant was added; i.p., intraperitoneal injection; i.m., intramuscular injection; PO, oral administration; *, relative percentage survival (RPS) calculating for vaccine efficacy (RPS = [1 minus vaccine group mortality/control group mortality] x 100).

## 7. Concluding remarks

*E. tarda* is a versatile Gram-negative bacterium that exhibits a broad geographical distribution and host range, and causes significant economic losses to the aquaculture industry. Despite limitations in this emerging field, recent studies on various virulence factors of *E. tarda* have enhanced our understanding of the pathogenesis of *E. tarda*, which adhere to, invade, and replicate in host cells and modulate their own gene expression to survive and adapt in fish. In addition, immune studies on edwardsiellosis applying proteomic and genomic approaches suggest that hosts sense the bacterium, induce inflammatory responses, and synergistically direct innate and adaptive immune responses against *E. tarda* infection. Furthermore, numerous studies on edwardsiellosis have reported highly efficacious vaccines, efforts that will encourage the development of a novel vaccine for use in aquaculture.

## Competing interests

The authors declare that they have no competing interests.

## Authors’ contributions

SBP drafted the manuscript, tables, and figure. TA and TSJ critically evaluated and revised the manuscript for important intellectual content. All authors have read and approved the final manuscript.
